# High Light Intensity Potentiates *Bacillus amyloliquefaciens* PMB05-Associated Plant Immunity and Bacterial Soft Rot Control in *Arabidopsis thaliana*

**DOI:** 10.3390/plants15192893

**Published:** 2026-09-22

**Authors:** Ai-Ting Li, Yun-Chen Tsai, Yun-Ching Tsai, Sabrina Diana Blanco, Hsin-Ying Chung, Tzu-Pi Huang, Yi-Hsien Lin

**Affiliations:** 1 Department of Plant Medicine, National Pingtung University of Science and Technology, Pingtung 912301, Taiwan; vivian12451245@gmail.com (A.-T.L.); ruby0109888@gmail.com (Y.-C.T.); yct@mail.npust.edu.tw (Y.-C.T.); sabrinablanco9779@gmail.com (S.D.B.); 2 Department of Tropical Agriculture and International Cooperation, National Pingtung University of Science and Technology, Pingtung 912301, Taiwan; 3 Department of Plant Industry, National Pingtung University of Science and Technology, Pingtung 912301, Taiwan; hsinying@mail.npust.edu.tw; 4 Department of Plant Pathology, National Chung-Hsing University, Taichung 402202, Taiwan; tphuang@nchu.edu.tw

**Keywords:** bacterial soft rot, light intensity, plant immunity, salicylic acid, sustainable agriculture

## Abstract

Bacterial soft rot, caused by *Pectobacterium carotovorum* subsp. *carotovorum*, affects numerous vegetable crops. Beneficial microbes like *Bacillus amyloliquefaciens* PMB05 offer a sustainable alternative to traditional copper- or antibiotic-based controls, which risks the development of pathogen resistance. *Bacillus amyloliquefaciens* PMB05 enhances disease resistance to soft rot by intensifying PAMP-triggered immunity (PTI). Although our previous research showed that PMB05-intensified PTI correlates with a reduced photosynthetic electron transport rate under high light conditions, it remains unclear whether increasing light intensity can strengthen PMB05-mediated immune signaling to further enhance disease resistance in plant leaves. In this study, *Arabidopsis* plants were grown under varying light intensities to address this question. Furthermore, expression of the type III elicitor, HrpN protein, was applied to evaluate the impact of PMB05 treatment on immune activation. HrpN acts as a PAMP that can trigger plant defense responses. The results revealed that higher growth light intensity was associated with PMB05-intensified PTI response by increasing reactive oxygen species generation but suppresses callose deposition. In addition, PMB05-facilitated PTI-mediated stomatal closure occurred more rapidly and tightly under high light, thereby restricting pathogen invasion into leaf tissues. Disease assays further confirmed that higher light intensity significantly reduced soft rot severity in *A. thaliana*, while the combination of high light and PMB05 treatment achieved superior control efficacy. Furthermore, PMB05-associated disease protection under high growth light was not detected in *sid2*, *npr1*, and *nahG* plants, indicating that intact SA-associated functions are required for the detectable protective phenotype under the conditions tested. These findings demonstrate that environmental light management can strategically potentiate immunity-intensifying microbes, establishing a novel paradigm for sustainable disease control through the coordinated manipulation of light conditions and microbial application.

## 1. Introduction

Bacterial soft rot, caused by *Pectobacterium carotovorum* subsp. *carotovorum* (Pcc), infects a wide range of economically important crops, including cabbage, pepper, and radish [1]. This pathogen secretes pectinolytic and cellulolytic enzymes that degrade plant tissues, leading to tissue maceration and facilitating rapid disease development [2]. Soft rot is particularly prevalent under high-humidity and high-temperature conditions, causing substantial damage that reduces crop quality and results in severe yield losses [3]. Moreover, its rapid field dissemination and latent infection capacity pose significant challenges for disease management [4]. Currently, chemical agents such as kanamycin, streptomycin, and oxine-copper are commonly used for disease control; however, their prolonged application raises concerns regarding the development of resistance and environmental impacts [5,6,7,8]. Consequently, developing sustainable and effective disease management strategies has become a priority in modern plant pathology.

Among sustainable control approaches, beneficial microorganisms offer a viable and promising alternative, with *Bacillus* spp. demonstrating successful applications in the management of various plant diseases [9,10,11,12]. Notably, *Bacillus amyloliquefaciens* PMB05 effectively suppresses bacterial soft rot and has been shown to control various plant diseases, including soft rot [11,13,14,15,16,17]. Subsequent studies revealed that its biocontrol mechanism involves amplifying PAMP-triggered immunity (PTI) responses upon the plant recognition of pathogen-associated molecular patterns (PAMPs). Specifically, PMB05 enhances PTI-associated defenses, including stomatal closure, reactive oxygen species (ROS) burst, and callose deposition [13,16,18], and activates MAPK signaling to intensify salicylic acid (SA)-mediated plant resistance [19,20].

Plant disease resistance often requires substantial energy input, underscoring the importance of photosynthesis-derived energy [21]. Göhre et al. demonstrated that PTI activation reduces non-photochemical quenching (NPQ), and mutations disrupting NPQ impair PAMP-induced SA-responsive defense gene expression [22]. Additionally, Wang et al. showed that PMB05-mediated PTI intensification suppresses the electron transport rate (ETR), with inhibition becoming more pronounced under higher light intensities [16]. These findings suggest that PTI activation redirects photosynthetic energy toward defense pathways, thereby enhancing resistance. Thus, light intensity and its effects on photosynthetic activity may influence plant disease susceptibility. Building upon our recent findings that high light intensity enhances *Bacillus amyloliquefaciens* PMB05-mediated plant immunity and bacterial wilt biocontrol [23], it remains unknown whether this environmental strategy extends to other bacterial diseases, such as soft rot, and whether it requires specific signaling pathways.

In this study, experiments were conducted using a standard growth chamber with a light intensity of 100 μmol m^−2^ s^−1^ as the control, 50 and 200 μmol m^−2^ s^−1^ designated as low- and high-light treatments, respectively. We examined PTI responses triggered by HrpN, a PAMP from the soft rot pathogen, under these varying light conditions, focusing on ROS generation, callose deposition, and stomatal closure. We further assessed the effects of PMB05 on immune signaling intensity and soft rot control across the three light conditions. Because PAMP recognition activates the MPK3/MPK6 branch of the MAPK cascade, which directly promotes ICS1-dependent salicylic acid (SA) biosynthesis and NPR1-mediated defense gene expression [24,25,26], we hypothesized that the light- and PMB05-dependent potentiation of immunity observed in this study may likewise depend on SA signaling. To test this, three *Arabidopsis* mutants disrupted at sequential steps of the SA pathway were employed: *sid2* (ICS1-deficient, blocking SA biosynthesis), *npr1* (SA-perception deficient), and *nahG* (SA-degrading). The use of these mutants allowed us to determine which step of SA signaling is specifically required for the light- and PMB05-dependent enhancement of disease resistance under high light conditions.

Collectively, these findings will clarify how light intensity optimizes PMB05-mediated PTI, SA signaling, and soft rot biocontrol, establishing an integrated environmental-microbial strategy for sustainable agriculture.

## 2. Materials and Methods

### 2.1. Plant Materials and Growth Conditions

*Arabidopsis thaliana* Columbia-0 (Col-0) wild type and salicylic acid (SA) pathway mutants, including *nahG* (expressing bacterial salicylate hydroxylase, which degrades endogenous SA) [27], *npr1-1* (the key SA activator required for SA perception and downstream defense gene expression) [28], and *sid2-2* (defective in ICS1, the isochorismate synthase required for pathogen-induced SA biosynthesis) [29] were obtained from the Arabidopsis Biological Resource Center (ABRC). Seeds were sown in pots filled with sterilized mixture composed of peat moss, perlite and vermiculite (*v*/*v*/*v* = 10:1:1). One week after germination, seedlings were individually transplanted to new pots and grown in walk-in growth chambers. Light intensities composed of 50% red and 50% blue wavelengths were set at 50, 100, or 200 μmol m^−2^ s^−1^ photosynthetic photon flux density (PPFD) using LED SUN LIGHT (Z9, Hipoint, Kaohsiung, Taiwan). In the soft rot disease control assays, an additional growth condition using standard fluorescent lamps was included as a reference. The light intensity was set to 100 PPFD, with red, blue, and green spectral components in an approximate ratio of 1:1:1.5. Plants were continuously cultivated under their assigned light conditions for four weeks under a 16 h light/8 h dark photoperiod at 25 °C. Four-week-old plants were used for all subsequent assays and were maintained under their respective growth-light conditions during treatment and post-treatment incubation, unless otherwise stated.

### 2.2. Bacterial Strains and Culture Conditions

*Bacillus amyloliquefaciens* PMB05 and *Pectobacterium carotovorum* subsp. *carotovorum* Ecc17 [16] were streaked on nutrient agar (NA) and incubated at 28 °C for 24–48 h to obtain pure cultures. For leaf invasion assays, a rifampicin-resistant derivative (*P. carotovorum* subsp. *carotovorum* Ecc17-R) was used, which stably grows on NA supplemented with 250 μg/mL rifampicin (NA-R).

### 2.3. Preparation of HrpN Protein Extract

An HrpN protein extract was prepared following established protocols [16]. Briefly, *Escherichia coli* BL21 harboring pET16b-HrpN was cultured on Luria–Bertani (LB) agar plate with 100 μg/mL ampicillin at 37 °C for 16–18 h. The bacterial culture was then transfer into fresh LB liquid medium containing 100 μg/mL ampicillin at a 1:50 dilution. After 4 h incubation, 1 mM isopropyl β-D-1-thiogalactopyranoside (IPTG) was added, followed by 16–18 h incubation. Cells were harvested by centrifugation, resuspended in 10 mM phosphate buffer (pH 7.0), sonicated, and heated at 100 °C for 10 min. The heat-treated lysate was centrifuged, and the resulting supernatant was collected as the HrpN protein extract. The supernatant was quantified and adjusted to 0.5 mg/mL for use in subsequent assays.

### 2.4. Disease Severity and Biocontrol Assays

Soft rot resistance of Col-0 plants grown under different light intensities was assessed via the foliar spray inoculation method [20]. Bacterial suspensions of *B. amyloliquefaciens* PMB05 and *P. carotovorum* subsp. *carotovorum* Ecc17 were prepared and adjusted OD_600_ to 0.3. For biocontrol treatments, equal volumes of PMB05 and Ecc17 suspensions were mixed immediately before spray inoculation. Control consisted of Ecc17 suspension prepared with sterilized water. The mixtures were evenly sprayed onto *Arabidopsis* leaves, and plants were incubated in sealed humidity chambers under original light conditions. Disease symptoms were scored 24 h post-inoculation (hpi) on a 0–4 scale (0: no symptoms; 1: 1–25% leaf area affected; 2: 25–50%; 3: 50–75%; 4: 75–100%). Disease severity (DS) was calculated based on the scales, DS = [(1 × N_1_ + 2 × N_2_ + 3 × N_3_ + 4 × N_4_)/(4 × N)] × 100%, where N_i_ is the number of plants in each category and N is the total number of plants. The assay on SA pathway mutants was performed only under high-light conditions. The DS for each treatment was calculated from ten plants, and the results were analyzed by repeating the experiment three times.

### 2.5. Reactive Oxygen Species (ROS) Generation and Callose Deposition Assays

To evaluate ROS generation and callose deposition upon plant immunity activation in leaves grown under different light intensities, *Arabidopsis* leaves were infiltrated with HrpN and/or *B. amyloliquefaciens* PMB05 following the established protocols [16]. Briefly, leaves from plants grown under varying light intensities were infiltrated with treatment mixtures. Prior to infiltration, HrpN protein extract, *B. amyloliquefaciens* PMB05 bacterial suspension, or 25 mM Tris-HCl buffer (pH 7.5) were mixed at a 1:1 volume ratio to generate four treatment mixtures: HrpN alone, PMB05 alone, HrpN+ PMB05 co-treatment, and Tris-HCl buffer control. The final HrpN concentration in HrpN-containing treatments was 0.5 mg/mL. ROS generation and callose deposition were analyzed at 1 and 8 h post-infiltration (hpi), respectively. Infiltrated leaf areas were excised into strips and destained in 75% ethanol to remove chlorophyll. Destained leaf strips were rinsed in phosphate buffer. For ROS detection, strips were stained with 20 μM H_2_DCFDA (2′,7′-dichlorodihydrofluorescein diacetate; Molecular Probes, Eugene, OR, USA). For callose deposition, strips were stained with 0.01% aniline blue (Sigma, St. Louis, MO, USA). Fluorescent signals were visualized using a fluorescence microscope (Leica Microsystems, Wetzlar, Germany) equipped with GFP (for ROS) and DAPI (for callose) filter cubes. The intensity of ROS generation and callose deposition was quantified using ImageJ software ver. 1.54j (https://imagej.net/ij/, accessed on 1 July 2024). All fluorescence images within each assay were acquired using identical microscope acquisition settings and were analyzed using the same ImageJ threshold settings. Each treatment included ten ROS or callose images as replicates. The experiment was repeated three times.

### 2.6. Stomatal Closure Analysis

To assess differences in stomatal closure rates upon plant immunity activation in leaves grown under varying light intensities, *Arabidopsis* plants were treated with HrpN and/or *B. amyloliquefaciens* PMB05 following established protocols [20]. Plants grown under different light intensities were pre-incubated in humidity chambers one day prior to analysis to ensure fully open stomata. Treatment mixtures were then evenly sprayed onto leaf surfaces. After treatment, plants were returned to their original light conditions, and leaf samples were collected at 15, 30, 60, and 90 min. Leaves were affixed to transparent adhesive tape to remove excess epidermal tissue, mounted on slides, and observed under a bright-field microscope. Stomatal images were captured, and stomatal aperture sizes were measured using ImageJ software. Each treatment included measurements from at least three plants, with 100 stomata measured per plant as replicates. For each treatment, one fully expanded leaf was sampled from each independent plant. The experiment was repeated three times.

### 2.7. Quantification of Soft Rot Bacterial Invasion in Leaves

To determine whether light intensity-mediated stomatal closure affects soft rot bacterial invasion, *P. carotovorum* subsp. *carotovorum* Ecc17-R population levels were quantified in spray-inoculated leaves [20]. Treatment mixtures were prepared as described for disease severity and biocontrol assays and sprayed onto *Arabidopsis* plants grown under different light intensities. At 24 h post-inoculation, leaves were excised, surface-sterilized in 1% sodium hypochlorite for 2 min, rinsed with sterile water, and blotted dry with paper towels (surface-sterilized samples). Non-surface-sterilized leaves were used for total bacterial quantification. Bacterial populations were quantified by grinding 1 cm^2^ leaf discs in 100 μL sterile water using microcentrifuge tubes. Serial dilutions (100 μL) were spread-plated onto NA-R medium using an automated spiral plater (WASP Touch, Bingley, UK). After 48 h incubation, colony-forming units (CFUs) were counted and back-calculated as log CFU/cm^2^. Each treatment included five leaf discs as replicates.

### 2.8. Statistical Analysis

All experimental data were analyzed using SPSS Statistics software version 25 (IBM Corp., Armonk, NY, USA) or Rstudio version 1.2.1335 (Boston, MA, USA). One-way analysis of variance (ANOVA) was performed to determine significant differences among treatments. Depending on data characteristics, post hoc analyses were conducted using Student’s *t*-test or Tukey’s Honestly Significant Difference (HSD) test. Differences were considered statistically significant at *p* < 0.05. A two-way ANOVA was used to evaluate the main and interaction effects of light intensity and PMB05 treatment on HrpN-induced ROS generation and callose deposition. Global post hoc comparisons across all treatment combinations were performed using Tukey’s HSD test. Differences were considered statistically significant at *p* < 0.05. The interaction analysis was limited to these early PTI-associated outputs and was not used to infer interaction effects on bacterial invasion or disease severity.

## 3. Results

### 3.1. Effects of Bacillus amyloliquefaciens PMB05 on HrpN-Induced Immune Responses in Plants Grown Under Different Light Intensities

To investigate the regulatory effects of *B. amyloliquefaciens* PMB05 on PTI signaling in *A. thaliana* grown under varying light intensities, ROS generation and callose deposition were analyzed following HrpN treatment. In untreated controls and PMB05-only treatments, ROS generation slightly increased with higher light intensity. HrpN treatment significantly enhanced fluorescence signals compared to controls in plants grown at 100 and 200 PPFD, but not at 50 PPFD (Figure 1A and Supplementary Table S1). Quantitative analysis revealed that HrpN induced a 2.8-fold increase in ROS fluorescence in 200 PPFD-grown plants relative to untreated controls (Supplementary Table S1). Co-treatment with *B. amyloliquefaciens* PMB05 further modulated these responses. In 50 PPFD-grown plants, PMB05 did not significantly enhance HrpN-induced ROS generation. However, plants grown at 100 and 200 PPFD exhibited significantly stronger HrpN-induced ROS bursts, with the most pronounced effect observed at 200 PPFD, representing a 1.6-fold increase over HrpN treatment alone. In the subsequent analysis of callose deposition, the results exhibited that increasing light intensity did not induce callose deposition in either the untreated control or the PMB05 alone treatment. Under HrpN treatment, callose-associated fluorescence was detected in plants grown at all light intensities, and cotreatment of PMB05 consistently enhanced these fluorescence signals (Figure 2A). However, as growth light intensity increased, the fluorescence intensity reflecting HrpN-induced callose deposition became progressively weaker. Quantitative analysis showed a similar trend. When comparing the fold changes in fluorescence intensity between HrpN/PMB05 co-treatment and HrpN alone at each light level, co-treatment at 50 and 100 PPFD increased callose-associated fluorescence by 1.49- and 2.00-fold, respectively (Supplementary Table S1). Under 200 PPFD, PMB05 still intensified HrpN-induced callose fluorescence by 1.38-fold; however, the relative fluorescence intensity of HrpN/PMB05 co-treated plants grown at 200 PPFD was only 17.8% of that observed in plants grown at 50 PPFD.

Two-way ANOVA confirmed a highly significant interactive effect between light intensity and PMB05 treatment on both HrpN-induced ROS accumulation (*F*_2, 174_ = 52.33, *p* < 0.001) and callose deposition (*F*_2, 264_ = 10.34, *p* < 0.001) (Supplementary Tables S2 and S3). When conducting global post hoc comparisons across all six HrpN-treated groups, ROS generation remained at basal levels under low and moderate light (50 and 100 PPFD), whereas high light intensity (200 PPFD) dramatically elevated HrpN-induced ROS generation, reaching its global maximum upon co-treatment with PMB05 (Figure 1B). Conversely, callose deposition exhibited an opposing global trend across light conditions: PMB05 significantly enhanced HrpN-triggered callose accumulation under low to moderate light, achieving the highest overall level at 50 PPFD and remaining significantly higher than HrpN alone at 100 PPFD (Figure 2B). Under high light (200 PPFD), this intensification effect was completely abolished, with callose deposition dropping to the lowest statistical grouping across all treatments.

### 3.2. Effects of Bacillus amyloliquefaciens PMB05 on HrpN-Induced Stomatal Closure in Arabidopsis thaliana Grown Under Different Light Intensities

To determine whether *Bacillus amyloliquefaciens* PMB05 modulates the dynamics of PTI-associated stomatal closure in *Arabidopsis* grown under different light intensities, stomatal aperture was monitored over time following HrpN treatment. Across all light regimes and treatments, stomata gradually closed within 60 min after application. Under 50 PPFD, HrpN treatment reduced stomatal aperture more rapidly than the Tris buffer control. However, co-treatment with PMB05 did not cause a further significant reduction in stomatal aperture compared with HrpN alone under this light condition (Figure 3A). In contrast, under 100 and 200 PPFD, HrpN not only promoted faster stomatal closure but also resulted in a tighter final stomatal aperture compared with the Tris control (Figure 3B,C). Co-treatment of PMB05 under these higher light intensities led to an even more pronounced decrease in stomatal aperture, with stomata significantly narrower than those in HrpN alone treatments at most time points examined. Microscopic observations at 15 and 60 min supported these quantitative data (Figure 3D,E). As growth light intensity increased, HrpN-induced stomatal closure became visibly faster, and the combination of HrpN with PMB05 resulted in stomata that were more tightly closed than in any other treatment under higher light intensities.

### 3.3. Effects of Bacillus amyloliquefaciens PMB05 on Soft Rot Bacterial Invasion in Arabidopsis Leaves Grown Under Different Light Intensities

To determine whether the dynamics of stomatal closure influence the invasion of soft rot bacteria, *A. thaliana* grown under different light intensities were spray-inoculated with *Pectobacterium carotovorum* subsp. *carotovorum* Ecc17-R, and both total and internal bacterial populations were quantified. At 24 h post-inoculation, total bacterial populations on leaf surfaces did not differ significantly among plants grown under different light intensities, and PMB05 treatment did not alter these values compared with the untreated controls (Figure 4). In contrast, analysis of internal bacterial populations revealed that, in the absence of PMB05, plants grown under different light intensities contained similar levels of invading Ecc17-R. PMB05 treatment effectively reduced pathogen invasion at all light levels, with internal bacterial populations in leaves of plants grown at 50 and 100 PPFD being 5.07 and 5.15 log CFU/cm^2^, respectively, showing no significant difference between these two conditions. Under 200 PPFD, PMB05 further decreased the internal bacterial population to 4.23 log CFU/cm^2^, representing a significant additional reduction compared with plants grown at lower light intensities.

### 3.4. Effects of Bacillus amyloliquefaciens PMB05 on the Control of Bacterial Soft Rot Disease in Arabidopsis Grown Under Different Light Intensities

To evaluate the role of *Bacillus amyloliquefaciens* PMB05 in disease control of bacterial soft rot under different light conditions, *A. thaliana* were grown either under standard white fluorescent light at 100 PPFD or under red–blue LED light at 50, 100, or 200 PPFD, and disease severity was assessed 24 h after spray inoculation with *Pectobacterium carotovorum* subsp. *carotovorum* Ecc17. Under the white-light condition, PMB05 treatment significantly reduced soft rot severity compared with the untreated control, with disease control efficacy reaching 61.9% (Figure 5). In the red-blue light regimes without PMB05, disease severity progressively decreased as growth light intensity increased, with values of 87.5%, 66.4%, and 44.1% for plants grown at 50, 100, and 200 PPFD, respectively. When PMB05 was applied, plants grown at 50 PPFD showed no significant reduction in disease severity compared with the corresponding untreated controls. In contrast, *A. thaliana* grown under higher red-blue light intensities exhibited greater reductions in disease severity upon PMB05 treatment, with disease severity at 100 and 200 PPFD decreased to 52.4% and 25.2%, corresponding to control efficacies of 21.0% and 42.8%, respectively.

### 3.5. Requirement of SA-Associated Functions for Early HrpN- and PMB05-Associated Stomatal Closure Under High Light

To assess the early phase of HrpN- and PMB05-associated stomatal closure, stomatal apertures were measured at 15 min after treatment in Col-0 and SA pathway defective plants grown at 200 PPFD. This time point was selected because PMB05-associated enhancement of HrpN-induced closure was clearly detectable in Col-0 plants at 15 min under 200 PPFD (Figure 3C). In Col-0 plants, the mean stomatal aperture in the untreated control was significantly reduced upon HrpN treatment, and co-treatment with PMB05 further enhanced stomatal closure, decreasing the aperture to approximately 1.6 μm (Figure 6). In contrast, *sid2-2*, *npr1-1*, and *nahG* plants showed a consistent loss of HrpN- and PMB05-induced stomatal closure. In all three mutants, stomatal apertures did not differ significantly among the Tris control, HrpN alone, and HrpN combining PMB05 treatments, indicating that neither HrpN nor PMB05 could promote stomatal closure in the absence of a functional SA pathway (Figure 6). Moreover, stomatal apertures in these SA pathway mutants were clearly larger than those in Col-0 under the corresponding treatments.

### 3.6. Requirement of Salicylic Acid Signaling for PMB05-Mediated Restriction of Soft Rot Bacterial Invasion Under High Light

To test whether the SA-associated functions are required for *Bacillus amyloliquefaciens* PMB05-mediated restriction of soft rot bacterial invasion observed under high growth light, total and internal populations of *Pectobacterium carotovorum* subsp. *carotovorum* Ecc17-R were quantified in *Arabidopsis* Col-0 and SA-related mutants grown at 200 PPFD following spray inoculation. The total bacterial populations recovered from leaves did not differ significantly between PMB05-treated and untreated plants in Col-0, *sid2-2*, *npr1-1*, or *nahG* plants (Figure 7). In contrast, analysis of internal bacterial populations revealed a distinct SA-dependent effect. In Col-0 plants, PMB05 treatment significantly reduced the internal population of Ecc17-R from approximately 6.15 to 3.92 log CFU/cm^2^. However, PMB05 treatment did not significantly reduce internal bacterial populations in the SA-related mutants *sid2-2*, *npr1-1*, or *nahG* (Figure 7), in which internal Ecc17-R populations remained between 5.39 and 6.26 log CFU/cm^2^.

### 3.7. Requirement of the SA Pathway for PMB05-Mediated Control of Bacterial Soft Rot Under High Light

To verify whether the enhanced resistance to bacterial soft rot conferred by *Bacillus amyloliquefaciens* PMB05 is dependent on the SA defense pathway, *Arabidopsis* Col-0 and SA-related mutants were grown at 200 PPFD and spray-inoculated with *Pectobacterium carotovorum* subsp. *carotovorum* Ecc17 in the presence or absence of PMB05. In Col-0 plants, disease severity reached 41.2% following pathogen inoculation and was significantly reduced to 22.8% by PMB05 treatment, corresponding to a control efficacy of 44.6% (Figure 8). In contrast, the SA-related mutants *sid2-2*, *npr1-1*, and *nahG* all developed disease severity exceeding 80% after Ecc17 inoculation. Moreover, PMB05 treatment did not produce a statistically detectable reduction in disease severity in any of the SA-pathway defective backgrounds under the inoculum density and disease-scoring conditions used (Figure 8).

## 4. Discussion

Bacterial soft rot caused by *Pectobacterium carotovorum* subsp. *carotovorum* (Pcc) is a destructive disease that rapidly macerates host tissues under warm and humid conditions, resulting in substantial crop losses [1]. Our previous studies demonstrated that *Bacillus amyloliquefaciens* PMB05 suppresses bacterial soft rot by potentiating PAMP-triggered immunity (PTI). PMB05-mediated PTI intensification was also associated with a reduction in photosynthetic electron transport rate (ETR), and this inhibitory effect became more pronounced as light intensity increased [16]. Because light is essential not only for photosynthesis but also for the regulation of stomatal behavior and plant–microbe interactions, manipulation of the light environment may provide an opportunity to optimize PMB05-mediated disease control. In our recent work on tomato bacterial wilt, increasing light intensity enhanced both PMB05-associated immune responses and disease control efficacy [23]. However, bacterial wilt is caused by a soil-borne pathogen that infect primary through the roots; thus, leaf-based assays do not directly model the primary root entry stage of bacterial wilt infection. In contrast, bacterial soft rot provides a suitable foliar disease system for evaluating how light intensity affects PMB05-mediated PTI, pathogen entry, and disease suppression.

In this study, *A. thaliana* grown under 50, 100, or 200 PPFD were treated with HrpN, a PAMP derived from the soft rot pathogen, either alone or in combination with PMB05. PMB05-enhanced ROS generation increased with growth light intensity and was most pronounced at 200 PPFD. In contrast, HrpN-induced callose deposition decreased as light intensity increased, although PMB05 enhanced callose-associated fluorescence relative to HrpN treatment alone within each light level. A comparable differential response was observed previously following treatment with the bacterial wilt pathogen-derived elicitor PopW [23]. Light availability alters cellular redox status and chloroplast-derived ROS signaling, whereas callose deposition is regulated by multiple elicitor-, hormonal-, and environmental factors [22,30,31,32]. Therefore, the contrasting responses of ROS and callose deposition suggest that increased light intensity differentially regulates distinct PTI outputs rather than uniformly enhancing all defense responses. The significant interaction between growth light intensity and PMB05 treatment for ROS generation and callose deposition indicates that growth light modifies PMB05-associated regulation of these early PTI outputs. However, because formal interaction analyses were not conducted for internal bacterial populations or disease severity, the present data do not establish statistical synergy for bacterial invasion or disease suppression. Instead, the disease assays show that PMB05-associated control efficacy was greater under higher growth-light conditions. Although ROS can promote callose deposition [19], the inverse ROS–callose pattern observed here indicates that these two PTI outputs are not obligatorily coupled during long-term growth light acclimation. Defense related callose deposition depends on callose synthase expression and activity, substrate availability, vesicle trafficking, callose turnover, and hormonal regulation [32]. High growth light acclimation may alter one or more of these processes, including the regulation of callose synthase related components; however, these possibilities were not directly tested in the present study. In addition to ROS generation and callose deposition, stomatal closure is an essential component of PTI that restricts the entry of foliar bacterial pathogens through natural openings. PMB05 has previously been shown to accelerate PTI-associated stomatal closure through a MAPK-dependent mechanism [20]. Here, PMB05 further accelerated HrpN-induced stomatal closure under high light, and increasing growth light intensity resulted in more rapid and tighter stomatal closure. This enhanced stomatal defense was associated with a marked reduction in the internal population of Pcc and improved suppression of bacterial soft rot. These findings indicate that high growth light conditions were associated with stronger PMB05-intensified ROS generation and rapid stomatal closure, which were accompanied by reduced pathogen invasion and disease development. The HrpN protein extract was used as a standardized elicitor to compare early PTI-associated outputs across growth light conditions. However, the responses induced by this extract should not be considered equivalent to the complete response elicited by live *P. carotovorum* subsp. *carotovorum* infection. Live Pcc carries multiple microbial patterns and additionally produces cell wall-degrading enzymes, effectors, and other virulence-associated factors that are not represented by the HrpN protein extract. The live-pathogen invasion and disease assays were therefore included to evaluate the biological relevance of the observed light- and PMB05-associated defense phenotypes. Moreover, *Bacillus* spp. can suppress plant pathogens through direct antagonism, including the production of lipopeptides and other antimicrobial metabolites. For example, strong direct antifungal activity mediated by lipopeptides has been reported for *Bacillus velezensis* TCS001 against *Magnaporthe oryzae* [33]. However, previous studies have characterized PMB05 as a comparatively weak antagonist relative to certain *Bacillus* strains while demonstrating its distinctive capacity to intensify PAMP-associated plant immune responses and disease resistance [13]. In the present study, PMB05 did not significantly alter the total Pcc population associated with leaf surfaces, whereas PMB05-associated restriction of internal bacterial populations and disease suppression was not detectable in SA-pathway defective plants. These observations support an important contribution of host defense to the PMB05-associated phenotype under the conditions tested.

We also observed that PMB05 provided greater control efficacy under the white fluorescent light than under the red–blue LED light. This difference suggests that spectral composition, in addition to PPFD, may influence PMB05-mediated disease suppression. Nagendran and Lee reported that tomato plants grown under red or green light showed reduced disease severity caused by *Pseudomonas cichorii*, with green light producing the strongest reduction in disease incidence under their experimental conditions [34]. The greater PMB05-associated control efficacy observed under white fluorescent light suggests that light quality may influence disease control. However, because the fluorescent and red–blue LED systems were not spectrally normalized beyond total PPFD, the present comparison cannot identify the responsible wavelength component. Controlled experiments using matched PPFD and defined spectral compositions will be required to determine whether and how individual wavelength components influence PMB05-associated immunity and disease control. The present study focused on elucidating the SA pathway underlying the high-light-enhanced immunity under defined red–blue LED conditions.

After establishing that high light enhanced PMB05-associated immune responses and soft rot control, we investigated whether SA signaling was required for this enhanced resistance. Our earlier work in the bacterial wilt indicated that PMB05-mediated disease control requires a functional SA pathway [23]. In addition, previous evidence indicates that elevated light conditions can enhance SA accumulation and thereby activate disease resistance [35]. We therefore assessed the contribution of SA biosynthesis, accumulation, and signaling to PMB05-mediated defense under high light using *sid2-2*, *npr1-1*, and *nahG* plants. In Col-0 plants grown at 200 PPFD, HrpN reduced stomatal aperture, and PMB05 co-treatment further enhanced this rapid closure response. In contrast, neither HrpN alone nor HrpN plus PMB05 induced stomatal closure in *sid2-2*, *npr1-1*, or *nahG*. The mutant analysis was restricted to the 15 min time point selected from the Col-0 kinetic assay. Therefore, it demonstrates a requirement for SA-associated functions in early stomatal closure but does not resolve the complete closing kinetics of the mutant backgrounds. Full time-course analyses will be required to determine whether later stomatal responses are also impaired. In the bacterial invasion assay, PMB05 also failed to reduce the internal population of Pcc in these SA-pathway defective backgrounds. These results demonstrate that SA functions, including ICS1-dependent SA biosynthesis, endogenous SA accumulation, and NPR1-dependent signaling, are required for PMB05-associated early stomatal defense and bacterial restriction phenotypes observed under high growth light. This interpretation is supported by previous studies showing that PAMP-induced stomatal closure is impaired in *Arabidopsis* mutants defective in SA biosynthesis or signaling, including *ics1*/*sid2*, *eds5*, and *npr1*. SA is therefore an important regulator of stomatal immunity, although it acts together with other guard-cell signaling components, including ABA, ROS, calcium, nitric oxide, and oxylipin pathways [36,37]. Moreover, Guan et al. demonstrated that NPR1 is required for systemic acquired stomatal immunity: the *npr1-1* mutant failed to close stomata upon pathogen challenge, resulting in increased bacterial entry into leaves [28]. Stomatal immunity is controlled by an interconnected guard-cell signaling network involving ABA, ROS, Ca^2+^, nitric oxide, ethylene, oxylipins, and SA. Although the present genetic analysis indicates that SA-associated functions are necessary for the early PMB05-associated stomatal response under high growth light, ABA-related signaling was not evaluated. Thus, our findings do not establish SA as the sole regulator of stomatal closure or exclude potential SA/ABA crosstalk. In addition, the present study did not quantify free or conjugated SA or determine the expression of SA-associated marker genes. Therefore, the mutant phenotypes establish a requirement for intact SA-associated functions but do not directly demonstrate that high growth light or PMB05 increases SA accumulation or SA-responsive gene expression. Together, these findings support the conclusion that SA-associated functions are required for the PMB05-enhanced early stomatal defense and restriction of bacterial invasion under high growth light conditions. Finally, PMB05 treatment did not produce a statistically detectable reduction in soft rot severity in *sid2-2*, *npr1-1*, and *nahG* plants under high light. Thus, even when high light enhances PMB05-associated PTI outputs, a functional SA defense pathway remains indispensable for translating these responses into effective resistance against bacterial soft rot. Because disease severity in *sid2-2* approached the upper limit of the rating scale, a ceiling effect may have reduced sensitivity for detecting a weak residual PMB05-associated protective effect. Assays using lower inoculum densities and additional assessment time points will be required to evaluate potential residual protection in highly susceptible mutant backgrounds.

Collectively, our results demonstrate that high light enhances PMB05-mediated resistance to bacterial soft rot by differentially regulating PTI outputs, promoting ROS accumulation and SA-dependent stomatal closure while attenuating callose deposition. The accelerated stomatal closure was associated with reduced bacterial entry into leaf tissues and improved disease control. Importantly, the loss of PMB05-mediated stomatal defense, restriction of bacterial invasion, and disease suppression in *sid2-2*, *npr1-1*, and *nahG* plants indicates that intact SA-associated functions are required for the PMB05-associated protection observed in plants grown under high light.

## Figures and Tables

**Figure 1:**
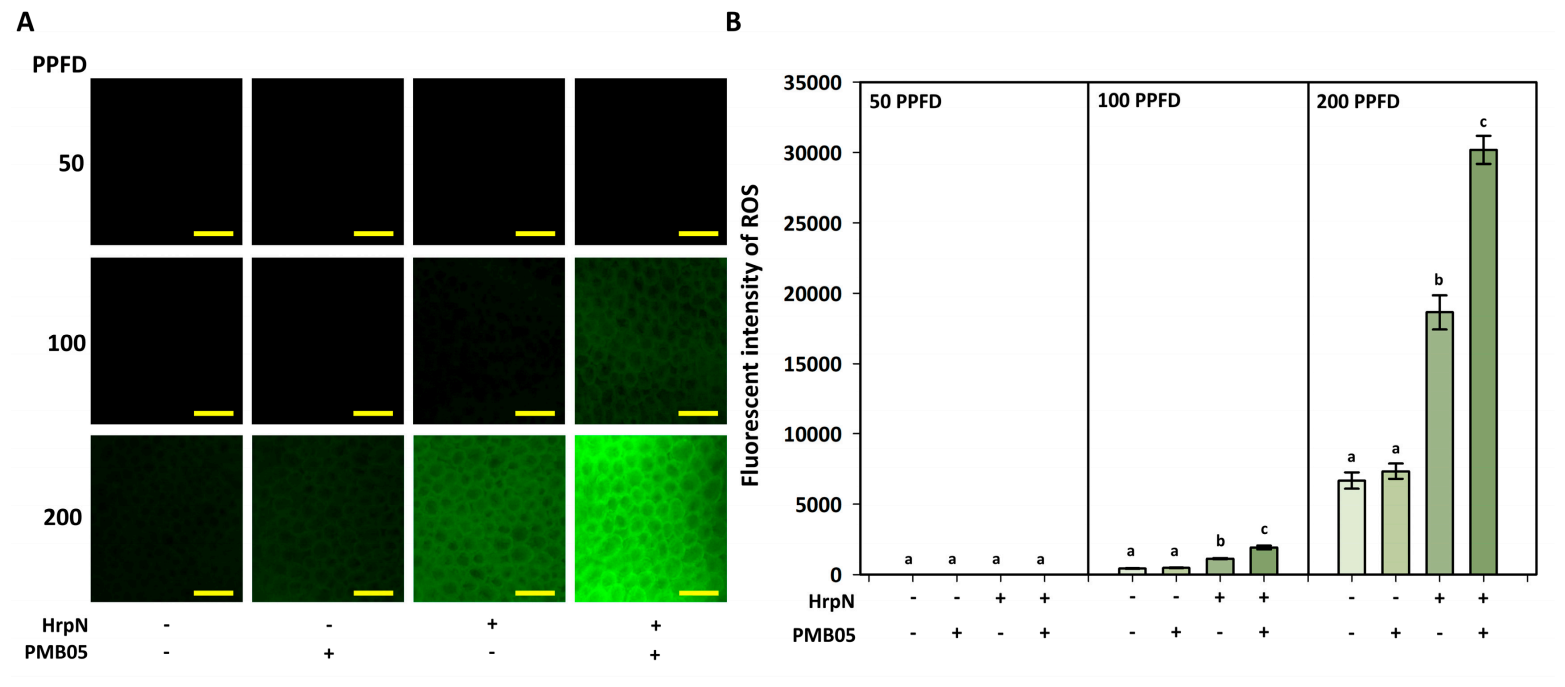
Effect of HrpN-mediated rapid reactive oxygen species (ROS) generation in *Arabidopsis thaliana* plants grown under different light intensities. Four-week-old *A. thaliana* Col-0 plants grown under 50, 100, or 200 PPFD were used in this assay. ROS generation was induced with 0.5 mg/mL HrpN protein extract in the presence (+) or absence (−) of *Bacillus amyloliquefaciens* PMB05. (**A**) Representative fluorescence images of ROS generation (scale bar = 20 μm). (**B**) Quantification of ROS fluorescence intensity. Data are presented as means ± standard deviations. Different letters indicate significant differences among treatments within each light intensity, as determined by Tukey’s HSD test (*p* < 0.05).

**Figure 2:**
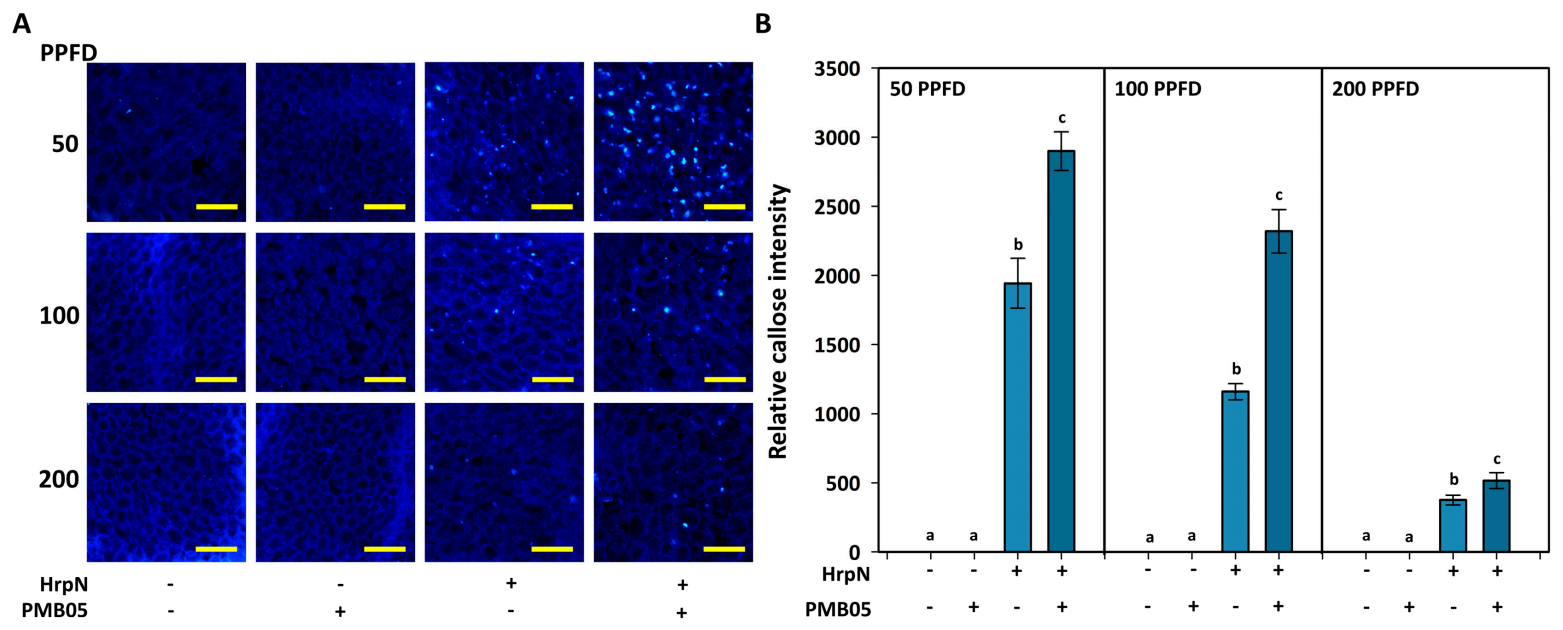
Effect of HrpN-mediated callose deposition in *Arabidopsis thaliana* plants grown under different light intensities. Four-week-old *A. thaliana* Col-0 plants grown under 50, 100, or 200 PPFD were used in this assay. Callose deposition was induced with 0.5 mg/mL HrpN protein extract in the presence (+) or absence (−) of *Bacillus amyloliquefaciens* PMB05. (**A**) Representative fluorescence images of callose deposition (scale bar = 20 μm). (**B**) Quantification of relative callose intensity. Data are presented as means ± standard deviations. Different letters indicate significant differences among treatments within each light intensity, as determined by Tukey’s HSD test (*p* < 0.05).

**Figure 3:**
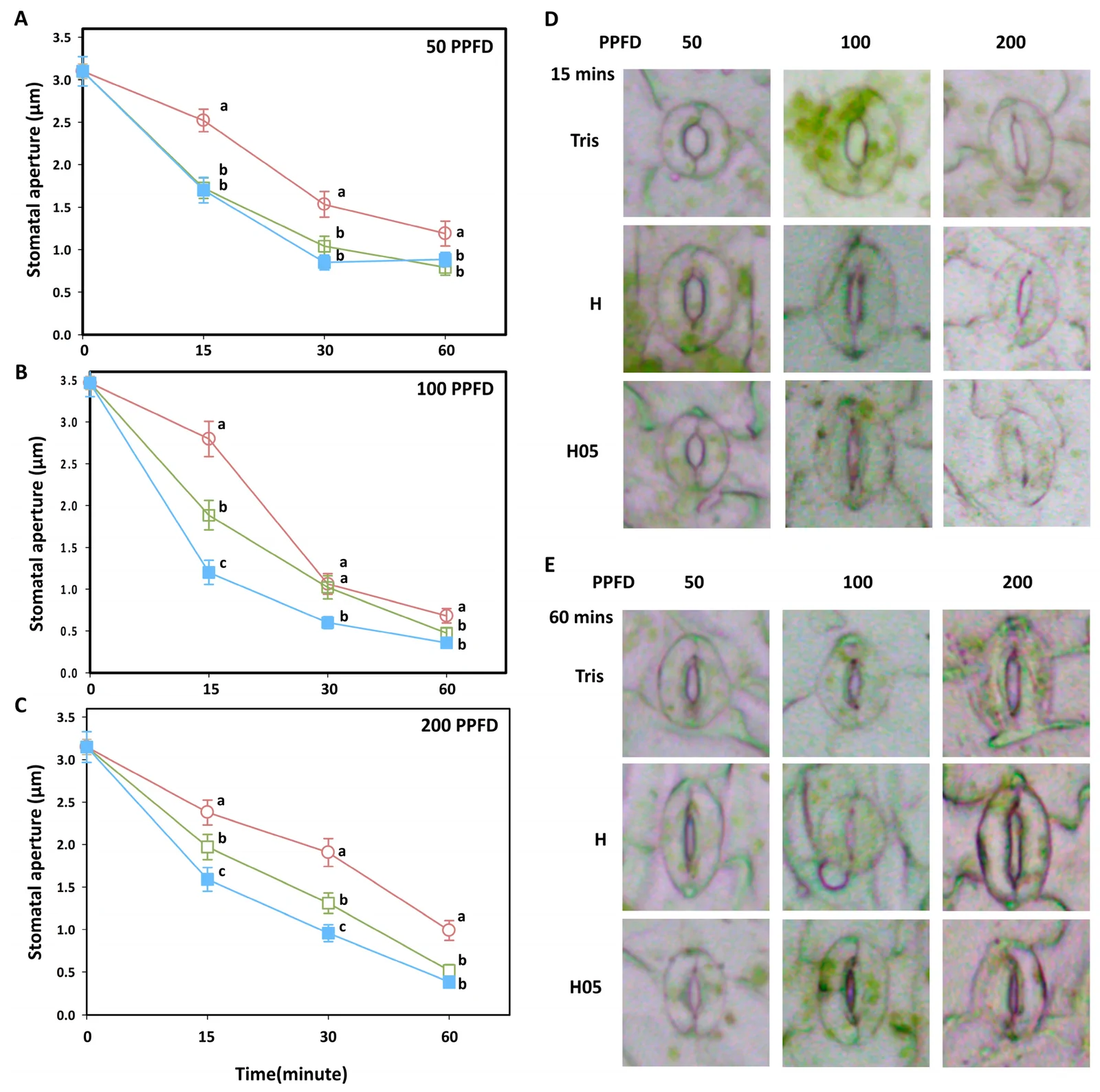
Effect of *Bacillus amyloliquefaciens* PMB05 on HrpN-induced stomatal closure in *Arabidopsis thaliana* plants grown under different light intensities. Four-week-old *A. thaliana* Col-0 plants grown under 50, 100, or 200 PPFD were treated with 25 mM Tris-HCl (pH 7.5) buffer (control, red open circles), 0.5 mg/mL HrpN protein extract alone (green open squares), or HrpN in combination with *B. amyloliquefaciens* PMB05 (blue filled squares), and stomatal apertures were measured at 0, 15, 30, and 60 min after treatment. (**A**–**C**) Time-course changes in stomatal aperture in plants grown under 50 PPFD (**A**), 100 PPFD (**B**), and 200 PPFD (**C**). (**D**,**E**) Representative images of stomata at 15 min (**D**) and 60 min (**E**) after treatment in plants grown under the indicated light intensities. Tris, buffer control; H, HrpN alone; H05, HrpN plus PMB05. Data are presented as means ± standard deviations. Different letters indicate significant differences among treatments at each time point, as determined by Tukey’s HSD test (*p* < 0.05).

**Figure 4:**
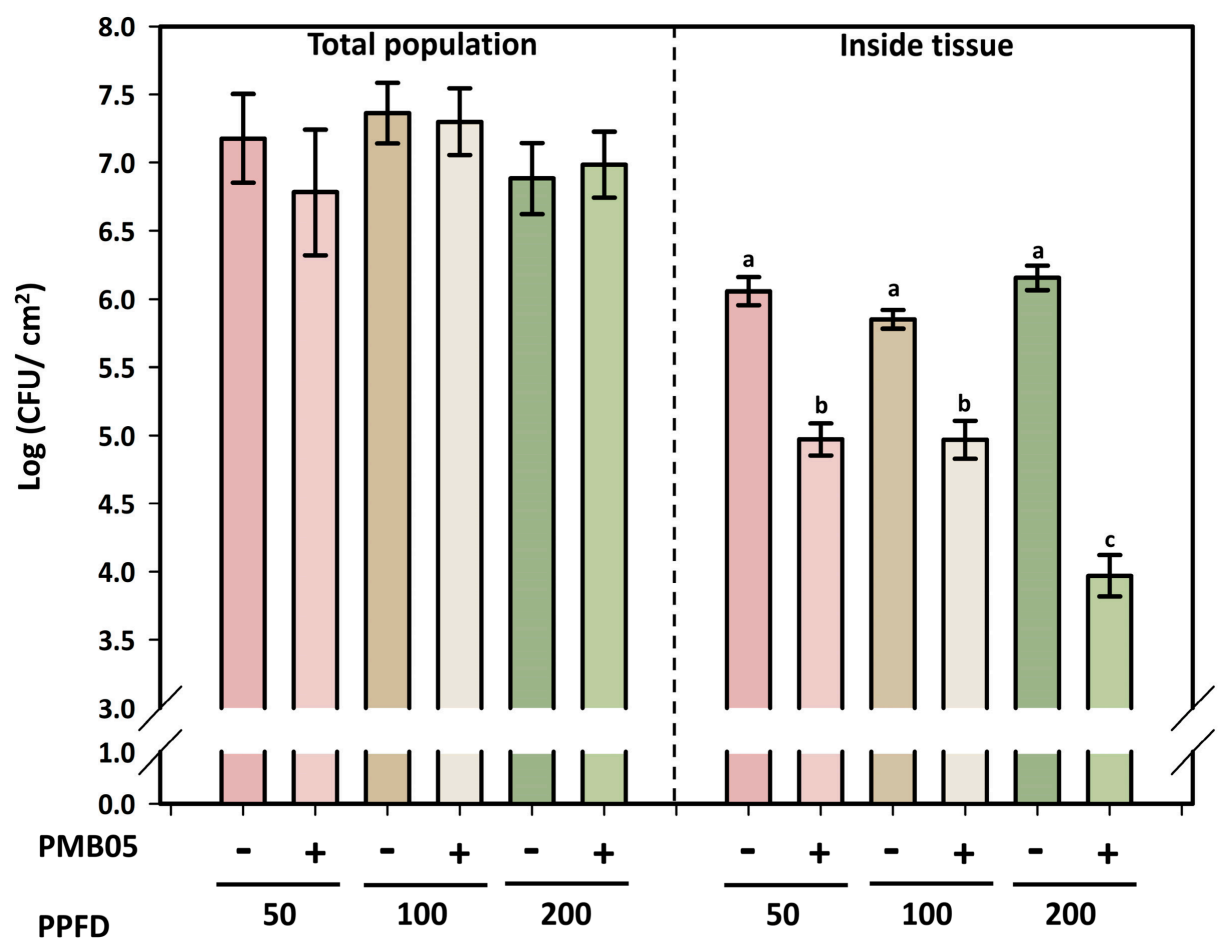
Effect of *Bacillus amyloliquefaciens* PMB05 on the invasion of *Pectobacterium carotovorum* subsp. *carotovorum* into leaves of *Arabidopsis thaliana* plants grown under different light intensities. Four-week-old *A. thaliana* Col-0 plants cultivated under 50, 100, or 200 PPFD were spray-inoculated with *P. carotovorum* subsp. *carotovorum* Ecc17-R in the presence (+) or absence (−) of *B. amyloliquefaciens* PMB05. At 24 h after inoculation, the total bacterial population associated with leaves (Total population) and the population recovered after surface sterilization (Inside tissue) were determined and expressed as log (CFU/cm^2^). Data are presented as means ± standard deviations. For the populations inside leaf tissue, different letters indicate significant differences among all treatments, as determined by Tukey’s HSD test (*p* < 0.05).

**Figure 5:**
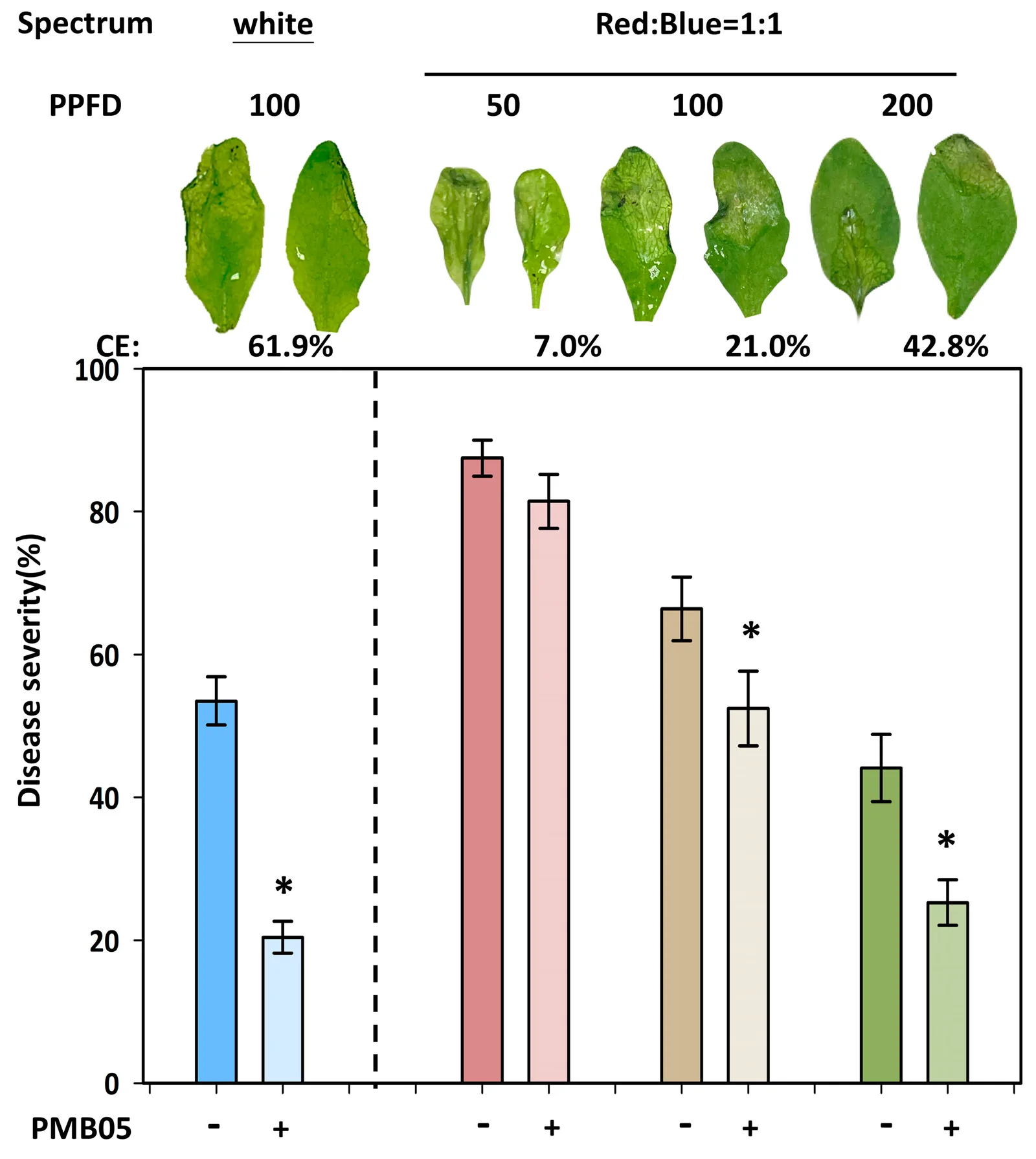
Effect of *Bacillus amyloliquefaciens* PMB05 on the control of bacterial soft rot in *Arabidopsis thaliana* plants grown under different light spectra and intensities. Four-week-old *A. thaliana* Col-0 plants were cultivated under white fluorescent light at 100 PPFD or under red–blue LED light (red:blue = 1:1) at 50, 100, or 200 PPFD, and then spray-inoculated with *Pectobacterium carotovorum* subsp. *carotovorum* Ecc17 in the presence (+) or absence (−) of *B. amyloliquefaciens* PMB05. Disease severity was assessed at 24 h after inoculation. Upper panels show representative disease symptoms of inoculated leaves; the corresponding control efficacy (CE) of PMB05 under each light condition is indicated above the graph. Data are presented as means ± standard deviations. Asterisks indicate significant differences between PMB05-treated plants and the untreated control under the same light condition (Student’s *t*-test, *p* < 0.05). The white fluorescent light treatment was included as a practical reference condition and was not intended as a spectrally normalized comparison with the red–blue LED treatments.

**Figure 6:**
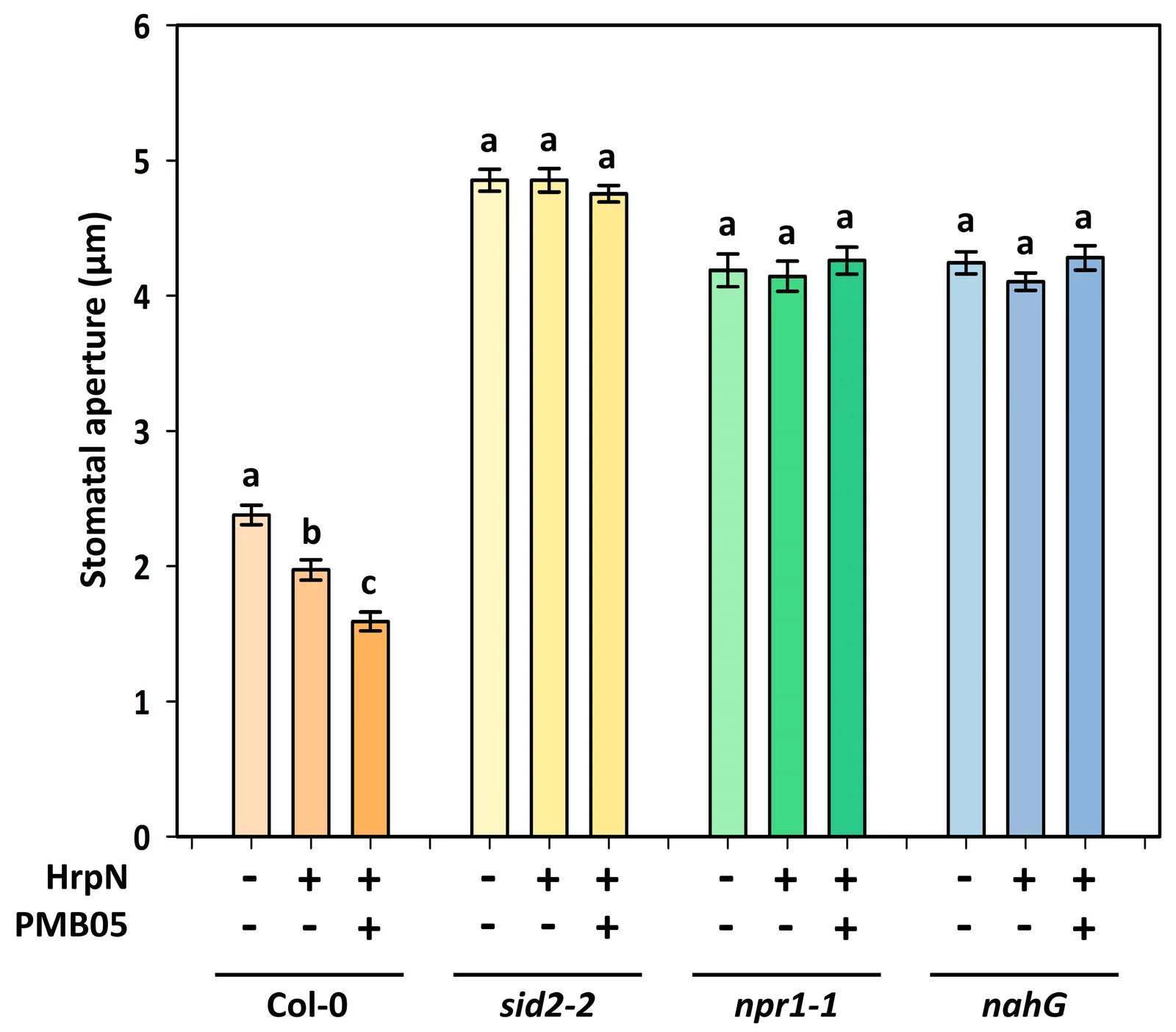
Effect of *Bacillus amyloliquefaciens* PMB05 on early HrpN-associated stomatal closure in *Arabidopsis thaliana* salicylic acid pathway mutants grown under high light. Four-week-old *A. thaliana* Col-0 and the salicylic acid (SA) pathway mutants *sid2-2*, *npr1-1*, and *nahG* were cultivated under 200 PPFD. Leaves were treated with Tris buffer (control), 0.5 mg/mL HrpN protein extract alone, or HrpN in combination with *B. amyloliquefaciens* PMB05, and stomatal apertures were measured at 15 min after treatment. The symbols “−” and “+” indicate the absence or presence of HrpN or PMB05, respectively. Data are presented as means ± standard deviations. Different letters indicate significant differences among treatments within each genotype, as determined by Tukey’s HSD test (*p* < 0.05).

**Figure 7:**
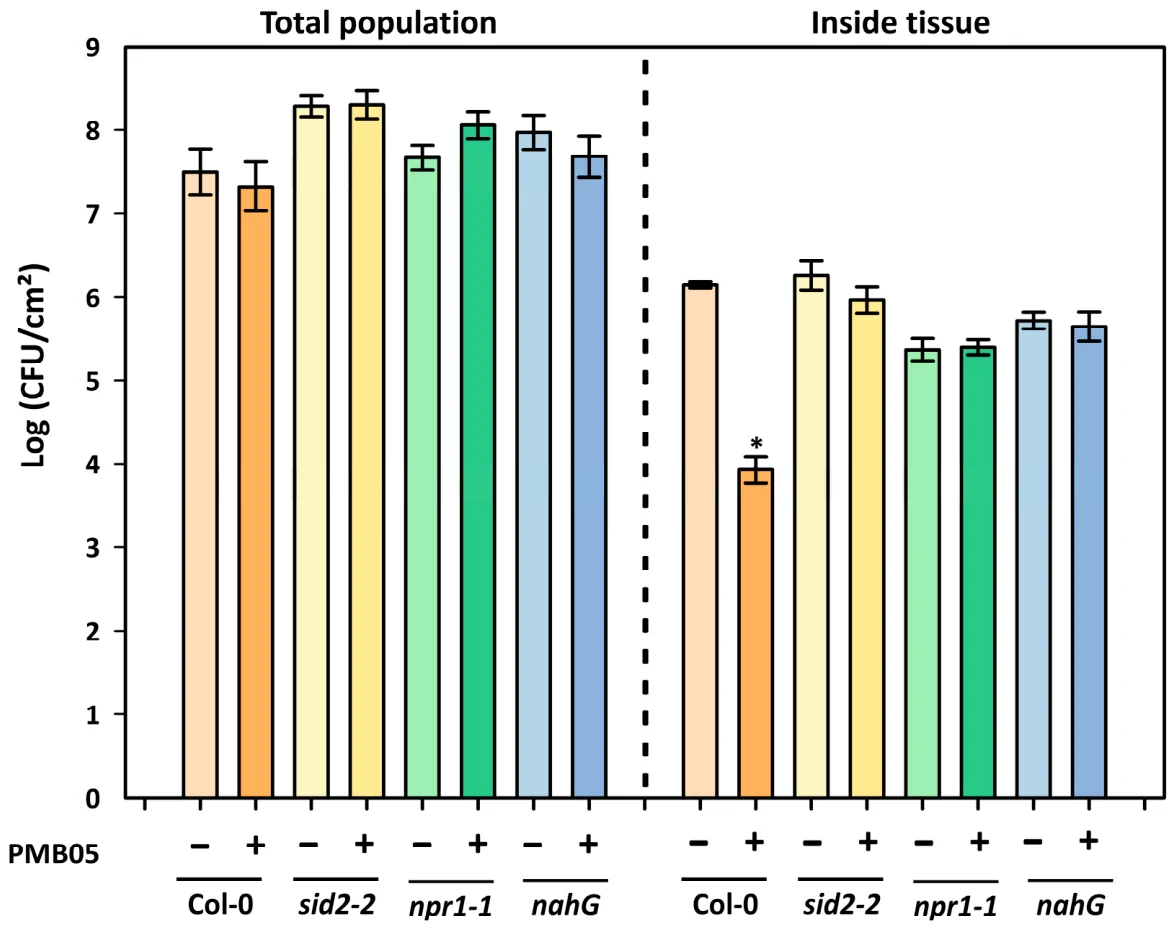
Requirement of the salicylic acid pathway for *Bacillus amyloliquefaciens* PMB05-mediated restriction of soft rot bacterial invasion in *Arabidopsis thaliana* plants grown under high light. Four-week-old *A. thaliana* Col-0 and the salicylic acid (SA) pathway mutants *sid2-2*, *npr1-1*, and *nahG* were cultivated under 200 PPFD and spray-inoculated with *Pectobacterium carotovorum* subsp. *carotovorum* Ecc17-R in the presence (+) or absence (−) of *B. amyloliquefaciens* PMB05. At 24 h after inoculation, the total bacterial population associated with leaves (Total population) and the population recovered after surface sterilization (Inside tissue) were determined and expressed as log (CFU/cm^2^). Data are presented as means ± standard deviations. Asterisks indicate significant differences between PMB05-treated plants and the untreated control within each genotype (Student’s *t*-test, *p* < 0.05).

**Figure 8:**
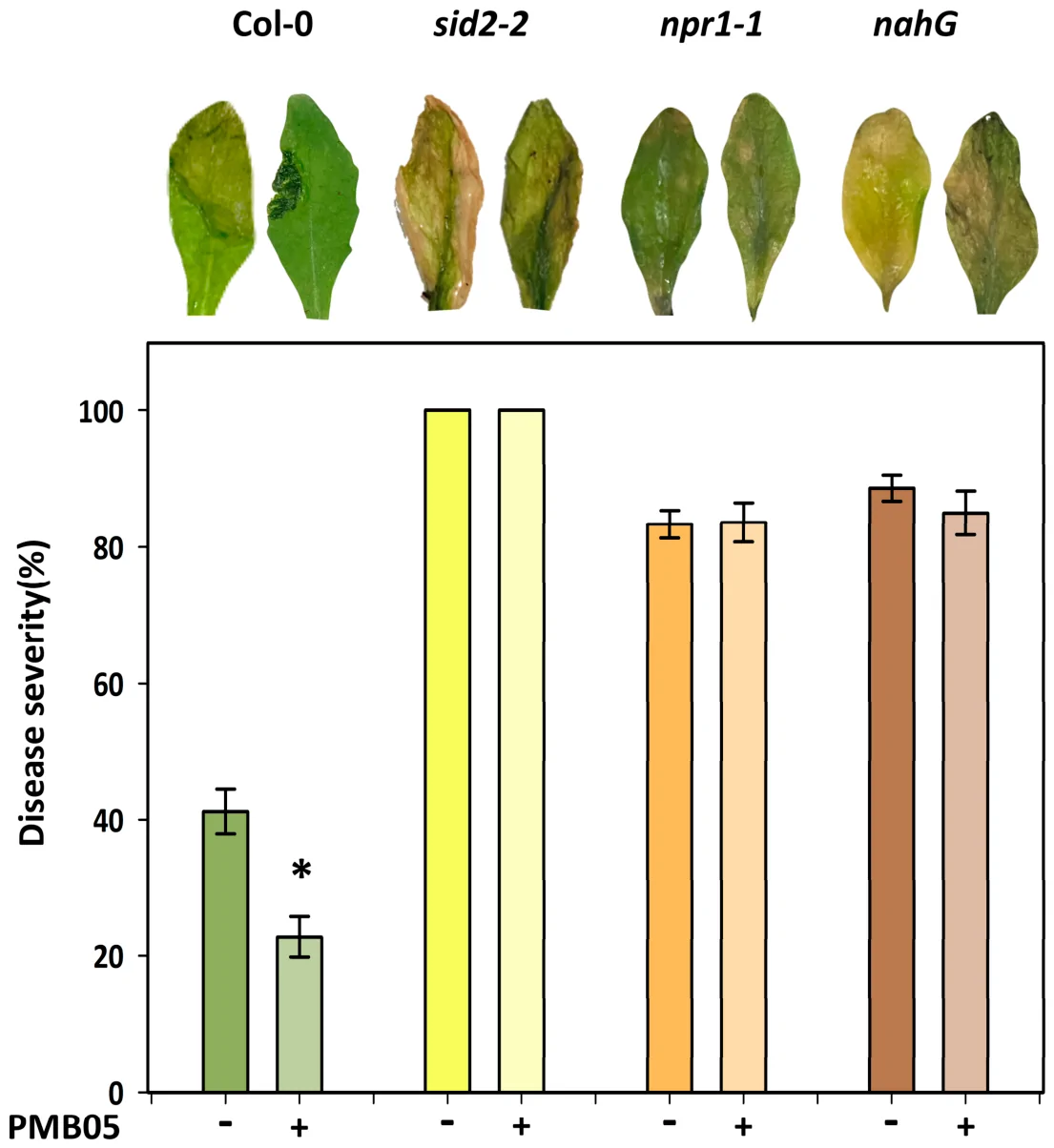
Requirement of the salicylic acid pathway for *Bacillus amyloliquefaciens* PMB05-mediated control of bacterial soft rot in *Arabidopsis thaliana* plants grown under high light. Four-week-old *A. thaliana* Col-0 and the salicylic acid (SA) pathway mutants *sid2-2*, *npr1-1*, and *nahG* were cultivated under 200 PPFD and spray-inoculated with *Pectobacterium carotovorum* subsp. *carotovorum* Ecc17 in the presence (+) or absence (−) of *B. amyloliquefaciens* PMB05. Disease severity was assessed at 24 h after inoculation. Upper panels show the representative disease symptoms of inoculated leaves. Data are presented as means ± standard deviations. Asterisks indicate significant differences between PMB05-treated plants and the untreated control within each genotype (Student’s *t*-test, *p* < 0.05).

## Data Availability

The original contributions presented in the study are included in the article; further inquiries can be directed to the corresponding author.

## Supplementary Materials

The following supporting information can be downloaded at: https://www.mdpi.com/article/10.3390/plants15192893/s1. Table S1. Effects of light intensity, *Bacillus amyloliquefaciens* PMB05, and HrpN elicitation on ROS fluorescent intensity and callose deposition in *Arabidopsis thaliana*; Table S2. Two-way ANOVA evaluating the main and interactive effects of light intensity and *Bacillus amyloliquefaciens* PMB05 treatment on HrpN-induced reactive oxygen species (ROS) burst in *Arabidopsis thaliana*; Table S3. Two-way ANOVA evaluating the main and interactive effects of light intensity and *Bacillus amyloliquefaciens* PMB05 treatment on HrpN-induced callose deposition in *Arabidopsis thaliana*.
