# Supplementary material for: High Light Intensity Potentiates Bacillus amyloliquefaciens PMB05-Associated Plant Immunity and Bacterial Soft Rot Control in Arabidopsis thaliana
Source: Plants (Basel). 2026 Sep 22;15(19):2893. doi: 10.3390/plants15192893 (PMC13666627; doi:10.3390/plants15192893)
Supplement: Supplementary file 1 [file plants-15-02893-s001.zip › plants-4571026-supplementary.pdf]

Supplementary Table S1. Effects of light intensity, *Bacillus amyloliquefaciens* PMB05, and HrpN elicitation on ROS fluorescent intensity and callose deposition in *Arabidopsis thaliana*.

| Light Intensity[x] | PMB05 | HrpN | ROS Fluorescent Intensity[z] | | Relative Callose Intensity[z] | |
|---|---|---|---|---|---|---|
| 50 | -[y] | - | 5.70 ± 2.18[z] | a | 0.09 ± 0.04 | a |
| | + | - | 0.27 ± 0.20 | a | 0.07 ± 0.04 | a |
| | - | + | 0.03 ± 0.03 | a | 1943.44 ± 180.23 | b |
| | + | + | 4.73 ± 2.28 | a | 2899.24 ± 139.89 | c |
| 100 | - | - | 424.90 ± 21.09 | a | 0.04 ± 0.03 | a |
| | + | - | 474.07 ± 22.53 | a | 0.04 ± 0.03 | a |
| | - | + | 1270.03 ± 38.08 | b | 1159.56 ± 57.45 | b |
| | + | + | 1567.87 ± 93.98 | c | 2319.44 ± 156.26 | c |
| 200 | - | - | 6690.20 ± 587.03 | a | 0.00 ± 0.00 | a |
| | + | - | 7353.03 ± 530.93 | a | 0.00 ± 0.00 | a |
| | - | + | 18642.53 ± 1211.89 | b | 374.98 ± 36.88 | b |
| | + | + | 30184.80 ± 1003.26 | c | 516.11 ± 56.02 | c |

[x] The unit of light intensity is $\mu$mol $m^{-2}$ $s^{-1}$ (PPFD, photosynthetic photon flux density).

[y] Tris-HCl was used as the vehicle control (-).

[z] Data are presented as Mean $\pm$ SE ($N = 30$). Different lowercase letters (a–c) within the same light intensity column indicate statistically significant differences based on one-way ANOVA followed by Tukey's HSD post-hoc test ($p < 0.05$).

Supplementary Table S2. Two-way ANOVA evaluating the main and interactive effects of light intensity and *Bacillus amyloliquefaciens* PMB05 treatment on HrpN-induced reactive oxygen species (ROS) burst in *Arabidopsis thaliana*.

| | Df[x] | Sum Sq[x] | Mean Sq[x] | F Value | Pr (>F) |
|---|---|---|---|---|---|
| Light[y] | 2 | 2.25E+10 | 1.13E+10 | 906.6 | < 2e-16 *** |
| PMB05 | 1 | 7.02E+08 | 7.02E+08 | 56.45 | 2.91E-12 *** |
| Light × PMB05 | 2 | 1.30E+09 | 6.49E+08 | 52.23 | < 2e-16 *** |
| Residuals | 174 | 2.16E+09 | 1.24E+07 | | |

[X] Df, degrees of freedom; Sum Sq, sum of squares; Mean Sq, mean square

[y] Light intensity levels evaluated were 50, 100, and 200 $\mu$mol $m^{-2}$ $s^{-1}$ PPFD

*** indicate statistical significance based on two-way ANOVA, $p < 0.001$

Supplementary Table S3. Two-way ANOVA evaluating the main and interactive effects of light intensity and *Bacillus amyloliquefaciens* PMB05 treatment on HrpN-induced callose deposition in *Arabidopsis thaliana*.

| | Df[x] | Sum Sq[x] | Mean Sq[x] | F Value | Pr (>F) |
|---|---|---|---|---|---|
| Light[y] | 2 | 181290554 | 90645277 | 143.43 | < 2e-16 *** |
| PMB05 | 1 | 38199349 | 38199349 | 60.44 | 1.70E-13 *** |
| Light × PMB05 | 2 | 13073977 | 6536989 | 10.34 | 4.73E-05 *** |
| Residuals | 264 | 166848185 | 632001 | | |

[X] Df, degrees of freedom; Sum Sq, sum of squares; Mean Sq, mean square

[y] Light intensity levels evaluated were 50, 100, and 200 $\mu$mol $m^{-2}$ $s^{-1}$ PPFD

*** indicate statistical significance based on two-way ANOVA, $p < 0.001$
